# Prevalence of Olfactory and Other Developmental Anomalies in Patients with Central Hypogonadotropic Hypogonadism

**DOI:** 10.3389/fendo.2013.00070

**Published:** 2013-06-07

**Authors:** Elisa Della Valle, Silvia Vezzani, Vincenzo Rochira, Antonio Raffaele Michele Granata, Bruno Madeo, Elisabetta Genovese, Elisa Pignatti, Marco Marino, Cesare Carani, Manuela Simoni

**Affiliations:** ^1^Unit and Chair of Endocrinology and Metabolism, Department of Biomedical, Metabolic and Neural Sciences, University of Modena and Reggio Emilia, Modena, Italy; ^*2*^Department of Medicine, Endocrinology, Metabolism and Geriatrics, Azienda USL of Modena, Modena, Italy; ^*3*^Department of Otolaryngology and Head and Neck Surgery, Azienda Ospedaliero-Universitaria Policlinico of Modena, Modena, Italy

**Keywords:** developmental anomalies, central hypogonadotropic hypogonadism, Kallmann syndrome, hypothalamic-pituitary-gonadal axis, neurosensorial hearing loss, midline defects, smell test

## Abstract

**Introduction:** Hypogonadotropic hypogonadism (HH) is a heterogeneous disease caused by mutations in several genes. Based on the presence of hyposmia/anosmia it is distinguished into Kallmann syndrome (KS) and isolated HH. The prevalence of other developmental anomalies is not well established.

**Methods:** We studied 36 patients with HH (31 males, 5 females, mean age 41.5), 9 with familial and 27 with sporadic HH (33 congenital, 3 adult-onset), by physical examination, smell test (BSIT Sensonics), audiometry, renal ultrasound, and magnetic resonance imaging of the olfactory structures.

**Results:** Based on the smell test, patients were classified as normosmic (*n* = 21, 58.3%) and hypo/anosmic (*n* = 15, 41.6%). Hypoplasia/agenesis of olfactory bulbs was found in 40% of patients (10/25; 75% hypo/anosmic, 7.6% normosmic, *p* < 0.01, Fisher’s test). Remarkably, olfactory structures were normal in two anosmic patients, while one normosmic patient presented a unilateral hypoplastic bulb. Fourteen of 33 patients (42.4%) presented neurosensorial hearing loss of various degrees (28.5% hypo/anosmic, 52.6% normosmic, *p* = NS). Renal ultrasound revealed 27.7% of cases with renal anomalies (26.6% hypo/anosmic, 28.5% normosmic, *p* = NS). At least one midline defect was found in 50% of the patients (53.3% hypo/anosmic, 47.6% normosmic, *p* = NS), including abnormal palate, dental anomalies, pectus excavatum, bimanual synkinesis, iris coloboma, and absent nasal cartilage. Anamnestically 4/31 patients reported cryptorchidism (25% hypo/anosmic, 5.2% normosmic, *p* = NS).

**Conclusion:** Hypo/anosmia is significantly related to anatomical anomalies of the olfactory bulbs/tracts but the prevalence of other developmental anomalies, especially midline defects and neurosensorial hearing loss, is high both in HH and KS and independent of the presence of anosmia/hyposmia. From the clinical standpoint KS and normosmic HH should be considered as the same complex, developmental disease.

## Introduction

Hypogonadotropic hypogonadism (HH) is characterized by a defective embryologic development of the Gonadotropin-releasing hormone (GnRH) pulse generator and the hypothalamo-pituitary-gonadal axis, with a prevalence from 1/4,000–1/10,000 in male and 1/40,000 in females (Grumbach, 2005). During normal embryonic development, the GnRH and olfactory neurons migrate together from the nasal olfactory epithelium to the basal hypothalamus, therefore a defect in this process may cause hypo/anosmic hypogonadism. The inability to perceive olfactory stimuli results from aplasia or hypoplasia of the olfactory bulbs and tracts. To date, hypogonadism associated with an olfactory deficit is defined as Kallmann syndrome (KS) and is distinct from normosmic HH (nHH), however both diseases share anatomical and genetic etiopathogenesis with common features. Many recent studies described several genes implicated in HH with different expressions also when the same mutation is present in different subjects within the same family (Pitteloud et al., 2006; Trarbach et al., 2006; Dodé et al., 2007), thus progressively thinning the border between KS and nHH. In fact, a recent study described mutations in genes previously thought to regulate primarily GnRH secretions in nHH (*GNRHR*, *TAC3*, *KISS1*, and *KISS1R*) in association with both normosmia and hyposmia; the precise mechanism is unclear but oligogenicity is a plausible reason (Lewkowitz-Shpuntoff et al., 2012). Previously, mutations in genes such as *FGFR1*, *FGF8*, *PROK2*, and *PROKR2* have been shown both in KS and in nHH, reinforcing the hypothesis of a pathophysiological overlap between these diseases. The majority of cases of HH are sporadic, but gene mutations or deletions can be detected in about 20% of cases of KS and in over 50% of cases of nHH, so that the old denomination “idiopathic” HH appears now obsolete (Simoni and Nieschlag, 2007). HH may be classified according to the severity of the pubertal defect as “complete,” if there is no evidence of puberty, or “incomplete,” if there is any evidence of sexual maturation (Burris et al., 1988). HH may also occur in adult age, commonly in the setting of infertility, in patients with completed puberty by the age of 18 years, so we can differentiate “congenital HH” and “adult-onset HH” (Nachtigall et al., 1997). HH generally requires lifelong therapy, however cases of spontaneous reversal later in life have been reported in about 10% of patients with either absent or partial puberty (Raivio et al., 2007). HH is a heterogeneous disease characterized not only by alteration of hypothalamo-pituitary-gonadal axis and/or olfactory structures but also by many developmental anomalies such as midline defects, hearing loss, renal anomalies, cryptorchidism. Only few studies reported the prevalence of these clinical features in KS patients, showing a large variability, and only rarely a comparison between KS and nHH was made (Quinton et al., 2001; Kaplan et al., 2010; Bonomi et al., 2012). In this study we assessed the phenotypical aspects of a normosmic and hypo/anosmic population of HH patients, demonstrating that, except for anatomical anomalies of the olfactory structures expectedly more frequent in hypo/anosmic patients, the other developmental defects are very common and are similarly found both in hypo/anosmic and normosmic patients.

## Materials and Methods

### Subjects

The study, partially prospective and partially retrospective, was approved by the local ethics committee and all subjects gave their written authorization to participation. From February 2009 to April 2012 we enrolled 36 patients, 31 males and 5 females (mean age 41.5), 9 with familial and 27 sporadic HH, 33 congenital, and 3 adult-onset, followed up at the Unit and Chair of Endocrinology and Metabolism of the University of Modena and Reggio Emilia, Italy. They fulfilled the following criteria: (1) absent or incomplete pubertal development by the age of 18 years, (2) low serum sex steroid levels with inappropriately low or normal gonadotropin levels (serum testosterone<100 ng/dl in men, primary amenorrea in women), (3) absence of other functional pituitary deficiencies or pituitary anomalies, (4) absence of causes of secondary hypogonadism. At the time of inclusion, 24 male patients were treated with androgenic replacement therapy, five male patients with gonadotropins, two men had withdrawn the therapy since many years (one because of spontaneous reversal HH and one because of poor compliance), three female patients were treated with estroprogestinic drugs, one woman was never treated, and one woman had stopped transdermic estrogens since many months.

### Methods

The patients were interviewed for medical, pharmacological and familial history, cryptorchidism, and pubertal development. Each patient underwent general physical examination, including searching middle line defects such us pectus excavatum, iris coloboma, absent nasal cartilage. Ogival palate, defined as high and narrow hard palate arch, cleft/lip palate, and absence of one or more teeth were assessed by clinical oral examination and anamnestically. Patients were asked to perform movements of one hand to assess the presence of involuntary synchronous mirror image movements in the opposite hand (bimanual synkinesis). In addition the following instrumental procedures were performed: audiometry in 33 patients, renal ultrasound in 36 patients (Esaote, MyLab ™70 XVision, Bio-Tek Instruments Inc.), nuclear magnetic resonance (NMR) of the olfactory structures in 25 patients (Philips Intera 3T Magnetic Resonance Imaging System). Sense of smell was tested by the Brief Smell Identification Test (BSIT Sensonics, NJ, USA), a microencapsulated odor “scratch and sniff” test with 12 items. Olfactory score of each subject was then interpreted using the age- and sex-related normative classification system described in the specific manual.

### Statistical analysis

For statistical analysis we used the software SigmaPlot (version 11.00 for Windows; Systat Software Inc., San Jose, CA, USA). Fisher’s exact test was used to determine statistical significance comparing prevalence of the anomalies between the hypo/anosmic group and normosmic group. All *p*-values are two-sided and *p* < 0.01 were considered significant.

## Results

### Smell and olfactory structures

Based on the smell test, patients were classified as normosmic (*n* = 21, 58.3%) and hypo/anosmic (*n* = 15, 41.6%). Hypoplasia/agenesis of olfactory bulbs was found in 10/25 patients (75% hypo/anosmic, 7.6% normosmic, *p* < 0.01) (Table 1): five bilateral and one unilateral hypoplasia, four bilateral agenesis. Remarkably, olfactory structures were normal in three anosmic patients, while one normosmic patient presented a unilateral hypoplastic bulb.

**Table 1:** **Percentages of developmental anomalies in HH patients**.

Developmental anomalies	Total patients	Hypo/anosmic HH	Normosmic HH	*P* (Fisher’s test)
Hypoplasia/agenesis of olfactory bulbs	40% (10/25)	75% (9/12)	7.6% (1/13)	0.001
Neurosensorial hearing loss	42.4% (14/33)	28.5% (4/14)	52.6% (10/19)	0.286
Renal anomalies	27.7% (10/36)	26.6% (4/15)	28.5% (6/21)	1
Midline defects (total)	50% (18/36)	53.3% (8/15)	47.6% (10/21)	1
Abnormal palate	44.4% (16/36)	40% (6/15)	47.6% (10/21)	0.741
Teeth agenesis	16.6% (6/36)	20% (3/15)	14.2% (3/21)	0.677
Supernumerary teeth	5.5% (2/36)	6.6% (1/15)	4.7% (1/21)	1
Pectus excavatum	5.5% (2/36)	6.6% (1/15)	4.7% (1/21)	1
Bimanual synkinesis	5.5% (2/36)	6.6% (1/15)	4.7% (1/21)	1
Iris coloboma	5.5% (2/36)	13.3% (2/15)	(0/21)	0.167
Absent nasal cartilage	5.5% (2/36)	13.3% (2/15)	(0/21)	0.167
Cryptorchidism	12.9% (4/31)	25% (3/12)	5.2% (1/19)	0.272

### Hearing

Fourteen of 33 patients presented neurosensorial hearing loss of various degrees (28.5% hypo/anosmic, 52.6% normosmic, *p* = NS) (Table 1), three cases unilateral, more frequently of medium-mild entity and in all cases for high frequencies. Of particular interest, we found bilateral absence of acoustic stapedius reflex in two hypo/anosmic patients and unilateral absence of acoustic stapedius reflex in one normosmic patient, not associated with hearing loss.

### Kidneys

Renal ultrasound revealed renal anomalies in 10/36 patients (26.6% hypo/anosmic, 28.57% normosmic, *p* = NS) (Table 1). In the normosmic group we found four cases of bilateral cysts, one case of unilateral hypoplasia, and one patient with hypertrophied column of Bertin (not considered a pathological feature but an anatomic variant); in the hypo/anosmic group we found two cases of unilateral agenesis, one case of bilateral cysts, and one case of unilateral nephrectomy because of renal cell carcinoma.

### Midline development

At least one midline defects was found in 18/36 patients (53.3% hypo/anosmic, 47.6% normosmic, *p* = NS): 16/36 abnormal palate, specifically 15 cases of ogival palate and 1 case of cleft-lip palate (40% hypo/anosmic, 47.6% normosmic, *p* = NS), 6/36 agenesis of one or more teeth (20% hypo/anosmic, 14.2% normosmic, *p* = NS) and 2/36 supernumerary incisors (6.6% hypo/anosmic, 4.7% normosmic, *p* = NS), 2/36 pectus excavatum (6.6% hypo/anosmic, 4.7% normosmic, *p* = NS), 2/36 bimanual synkinesis (6.6% hypo/anosmic, 4.7% normosmic, *p* = NS), 2/36 iris coloboma, and absent nasal cartilage (13.3% hypo/anosmic, 0% normosmic, *p* = NS). The latter anomalies were present together in two hypo/anosmic patient (one unilateral and one bilateral coloboma) and the patient with bilateral coloboma presented also other particular features: blepharophimosis, dystopia cantorum, oblique eye, microphthalmia, flat occipite, prognathism, bilateral clinodactyly of the fifth finger, bilateral elbow valgus.

### Cryptorchidism

None of the patients was currently cryptorchid. Anamnestically 4/31 patients reported having been treated for cryptorchidism (25% hypo/anosmic, 5.2% normosmic, *p* = NS), bilateral in three cases and treated with surgery, and unilateral in one case treated pharmacologically. Concerning the female gonads, we found a unilateral ovarian agenesis in one normosmic patient.

## Discussion

Before the discovery, in the last years, of the many genes implicated in the pathogenesis of HH, some studies, designed to compare the overall severity of HH between KS and nHH patients, suggested a more severe reproductive phenotype in anosmic patients, without taking into account the clinical heterogeneity of the disease. Recent studies compared the genotype-phenotype relationships (Salenave et al., 2008; Brioude et al., 2010; Sarfati et al., 2010), but to date the prevalence of clinical features of HH, especially of non-reproductive and non-olfactory anomalies, has not been reported in detail. Although in our study the relative small number of patients precludes really meaningful comparison between the two subgroups of patients, some comparison with the available scientific literature is possible. At odds with our results, Quinton et al. (2001) conducted the clinical evaluation of a large patient sample (170 males and 45 females) finding a phenotypic separation between KS and nHH, even if this study lacked statistical analysis. In this study, mirror movements and neurosensorial deafness were observed only in KS patients, with a percentage of 31 and 8% respectively. Conversely, in our population, these anomalies were present in both groups and hearing defects were even more frequent in normosmic than in hypo/anosmic patients although without significant difference. Quinton et al. (2001) also described a prevalence of cryptorchidism nearly three times greater in KS than in nHH, despite comparable testicular volumes. As regards this anomaly in our patients, we found no significant difference between the two groups. Noteworthy, the prevalence of cryptorchidism in normosmic patients was similar to that reported in a study of a large sample of 315 HH patient in both KS and nHH patients (Bhagavath et al., 2006), and the prevalence in hypo/anosmic patients was similar to that reported in a study of Kaplan et al. (2010) in five KS patients. In a recent study, Bonomi et al. (2012) found a prevalence of cryptorchidism of 35% in KS patients and 10% in nHH patients. Pitteloud et al. (2002) reported an higher prevalence of cryptorchidism (40%) in HH patients with absent pubertal development compared with patients with some evidence of prior pubertal development (5%). We cannot compare our results to this study because our subjects were not classified according to complete or incomplete HH, since at the time of evaluation, many patients had been treated with testosterone or gonadotropins for many years.

The prevalence of at least one midline defect in our study group was high (50%) and nearly equal between hypo/anosmic and normosmic patients, while in the study of Bonomi et al. (2012) this was only 6.6% and higher in KS than in nHH patients. Abnormal palate was very frequent in both groups of our study, mainly ogival palate, however cleft-lip palate, iris coloboma, and absent nasal cartilage were found only in hypo/anosmic HH. The rarity of cleft-lip palate was in line with previous studies (Kaplan et al., 2010). We found absence of at least one tooth (lower molars and wisdom teeth) in both group without significant difference. In the literature, dental agenesis was reported in KS with a prevalence of 5–10% (Bailleul-Forestier et al., 2010), lower than the prevalence in our hypo/anosmic patients, and was rarely reported in nHH (Pitteloud et al., 2006). In addition, we found two cases of supernumerary incisors not described in association with HH so far, although these data were not assessed by orthopantomograms. Some studies reported bimanual synkinesis only in KS with different prevalence ranging from 4.1% (Bonomi et al., 2012) to 31% (Quinton et al., 2001), while we reported this anomaly in both group without significant difference.

We found high prevalence (47% of total patients) of neurosensorial hypoacusia of different degrees, especially medium-mild, bilateral absence of acoustic stapedius reflex in two hypo/anosmic patients and unilateral absence of acoustic stapedius reflex in one normosmic patient, not associated with hearing loss. The latter anomaly may reflect a neuronal or mechanical alteration and requires further examinations to define the cause. The prevalence of hearing loss in HH was reported to date only in KS with high variability between several studies ranging from 2.1 to 40% (Kaplan et al., 2010; Bonomi et al., 2012). Our study is in line with the literature, with a prevalence of 28.5% in hypo/anosmic subjects, and demonstrates, in addition, that this anomaly can be found also in normosmic patients, although without significant difference.

Concerning renal anomalies, radiological studies of subjects with X-linked form of KS have shown that up to 40% of patients present an absent kidney unilaterally (Kirk et al., 1994), but also other anomalies have been reported such as urinary-tract duplication (Wegenke et al., 1975), hydronephrosis (Zenteno et al., 1999), renal diverticulum (Lieblich et al., 1982), horseshoe or rotated kidney (AbuJbara et al., 2004), multicystic dysplastic kidney, and vesicoureteral reflux (Sato et al., 2004). Bonomi et al. (2012) reported a much lower prevalence of renal agenesis/aplasia in KS (3.1%) and also described a case of renal agenesis in a nHH patient. In our hypo/anosmic group, the prevalence of kidney anomalies was 26.6%, similar to the data reported in the literature (Sato et al., 2004; Kaplan et al., 2010), but we found only two2 cases of renal agenesis. In the total population, this prevalence was nearly 28% without significant difference between the two groups. Interestingly ultrasound revealed five cases of bilateral cysts, more frequent in normosmic patients. Deeb et al. (2001) described two cases of antenatally diagnosed multicystic kidneys in two brothers with KS features but without *KAL1* mutations. Anosmin-1, encoded by *KAL1*, immunolocalizes early in the ureteric bud brunches and has a role in mediating cell adhesion during growth of the mesonephric duct/ureteric bud lineage. Since dysplastic renal tubules are poorly branched derivatives of the ureteric bud that terminate in cystic dilations, it is supposed that *KAL1* mutations can generate renal dysplasia as well as agenesis. However, cases of renal malformations are reported in members of families with KS individuals with normal *KAL1* genotype (Colquhoun-Kerr et al., 1999), suggesting the involvement of other genes. Multicystic kidneys tend to involute prenatally or in early childhood, therefore cases of renal agenesis detected in older patients may originate as multicystic dysplastic kidneys which subsequently regress. These malformed organs are often surgically removed because of the risk of hypertension and renal tumor development. In our hypo/anosmic population, we found one case of nephrectomy for renal carcinoma in a patient with several features of the Charge Syndrome (cleft-lip palate, pectus excavatum, unilateral iris coloboma, absent nasal cartilage, medium-severe neurosensorial hypoacusia, unilateral cryptorchidism); according to our knowledge this is the first case of a renal cell carcinoma in a HH patient.

Koenigkam-Santos et al. (2011) recently showed that olfactory bulb and sulcus aplasia were the most common findings in KS patients and demonstrated agreement between MRI findings and the smell test, especially the presence of bulb aplasia and anosmia were consistent. Therefore, MRI of olfactory structures is important to confirm the results of smell test, even if normal olfactory bulb images have been reported in a few KS patients (Vogl et al., 1994; Quinton et al., 1996) and, conversely, one normosmic subject showed a hypoplastic left olfactory bulb (Lewkowitz-Shpuntoff et al., 2012). This disagreement between the MRI findings and the smell test was present also in our study. In fact we described three hypo/anosmic patients with normal olfactory structures and without evidence of an upper airway acquired anomaly so that other possible causes of reduced sense of smell exist. Also we assume that the only normosmic subject with unilateral hypoplastic olfactory bulb may have a mild hyposmia not revealed with the smell test, or that the contralateral bulb compensates the hypoplastic bulb.

## Conclusion

Olfactory defects are significantly related to anatomical anomalies of the olfactory bulbs/tracts but the prevalence of other developmental anomalies, especially midline defects and neurosensorial hearing loss, is high both in nHH and KS independently of the presence of anosmia/hyposmia. From the clinical standpoint KS and nHH should be considered as the same complex disease. As a matter of fact, recently hypo/anosmic patients with mutations in genes previously associated only with nHH are reported in literature (Lewkowitz-Shpuntoff et al., 2012), confirming that KS and nHH are two different expression of the same pathology. Anosmia/hyposmia in the presence of apparently normal olfactory bulbs is intriguing and suggests that obviously other causes for this symptom must exist. In this respect the recent description of previously unrecognized, specific anomalies of the ethmoid bone is interesting (Maione et al., 2013). Further studies with a larger sample of patients need to confirm our results and to find other anomalies associated with this syndrome. Finally, detailed genetic analysis will allow precise genotype-phenotype characterization.

## Conflict of Interest Statement

The authors declare that the research was conducted in the absence of any commercial or financial relationships that could be construed as a potential conflict of interest.
